# What ethical AI and robots could mean: a conceptual and ethical reflection

**DOI:** 10.3389/frobt.2026.1769361

**Published:** 2026-04-13

**Authors:** Jana Sedlakova

**Affiliations:** Nuffield Department of Population Health, Ethox Centre, University of Oxford, Oxford, United Kingdom

**Keywords:** AI ethics, human-AI collaboration, human-AI interaction, relational ethics, sociotechnical systems, interdisciplinarity

## 1 Introduction

Recent advances in robotics and artificial intelligence (AI) are reshaping assumptions about moral agency, and moral or ethical interaction. The moral sphere is being expanded to non-human artificial agents. While in history, we expanded the moral sphere to non-human agents such as animals, it is the first time we expand it to something artificially created by us. AI and robots enter and co-shape our epistemic, social, ethical and professional spaces, and create new forms of collaboration and relationships. This raises a fundamental conceptual challenge: before we ask what makes robots or AI ethical or conscious, we must clarify what these terms mean in this novel context, and what assumptions shape the debates and ethical evaluation themselves.

This paper argues that the ethics of AI and robots requires moving beyond human-centered analogies and instead developing a framework that understands AI and robots on their own terms, acknowledges their strengths and limitations, and situates ethics within broader sociotechnical systems of meaning, values, and relationships. While the core arguments apply to both AI systems and robots, a closer ethical analysis requires distinguishing between these two (Küçükuncular, 2026). Within the scope of this opinion piece, I focus primarily on AI systems, while indicating where robots introduce distinct considerations, particularly through embodiment.

## 2 The need to move beyond the humanization narrative

Much of the contemporary debate, both public and academic, is shaped by a humanization narrative (Salles et al., 2020; Placani, 2024; Sedlakova, 2024b; 2024a). AI systems are described as simulating human cognition, empathy, or reasoning, or even exceeding human abilities and becoming conscious. Questions such as “Is AI conscious?” or “Is AI empathetic?” have a prominent place in discussions of ethical AI. While intuitively appealing, these questions risk obscuring more than they reveal by framing AI primarily through a human lens.

Humanization seems to be a powerful narrative tool. It can help to engage with complex technologies and provide intuitive anchors for ethical concern. However, it also introduces significant systematic challenges that motivate the need to move beyond the humanization narrative. In particular, I focus on three risks: 1) a normative gap between simulated and moral capabilities, 2) shifts of meaning of key concepts when applied to AI, and 3) methodological problems in evaluating AI when using human-centered benchmarks. Together, these challenges can create misleading expectations about AI’s role and its capabilities, and narrow the space of possible AI understandings, its design choices and ways of collaboration.

When AI is described as “listening without judgement,” “understanding,” or “caring,” these descriptions often refer to simulated conversational or behavioral patterns, not to experiential or moral capacities. This creates a normative gap: while AI simulates human abilities like empathy or trust, it cannot fulfil the moral requirements necessarily connected with these, such as being responsible (Sedlakova, 2024b; Sedlakova et al., 2025). While AI could be ethical by aligning its interaction with our values and principles, it is problematic to see it as a moral agent. Moral agency requires, for example, moral understanding, consciousness, responsibility, and lived experiences, which AI lacks (Véliz, 2021). The normative gap can lead to wrong expectations from the human-like AI, for example, perceiving it as having high authority. In clinical or mental health settings, conversational AI communicating in a confident, empathetic, and human-like manner may be perceived as a competent advisor. Users may follow its recommendations even when they are false or inappropriate because the system’s interaction style signals understanding and authority that it does not possess.

When we attribute human concepts to AI and interact with it, there is always a change of meaning (Coeckelbergh, 2021; Sedlakova et al., 2025). Trust between a psychotherapist and a client is different from trust between AI and a client. For example, trusting a therapist means that a therapist is responsible for their behavior, can justify their actions and apologize. Can AI apologize? Is it meaningful when it does? AI functions differently from humans (Bender et al., 2021; Felin and Holweg, 2024). AI systems operate within architectures of statistics, prediction, optimization, and classification. Their computational power and strength could be hidden behind the human-like conversational or interaction design which can lead to misplaced ethical evaluation and its focus, for example, on human metrics. Even though AI systems can support ethical outcomes without being moral agents, clarity about their role in relation to ethical requirements is necessary. Recognizing the importance of the distinction between AI systems and humans helps avoid both over-attribution of responsibility to AI systems and under-recognition of human responsibility in design, deployment, and governance. This difference should also be seen as an opportunity for creating novel ways of interaction that might not be enabled if the focus is overly on the simulation of humans. If we rely too much on the humanization narrative, alternative ways of imagining ethical interaction and collaboration remain underexplored. For example, understanding of AI in an interaction and collaboration that emphasizes complementarity rather than imitation, and coordination rather than equivalent capabilities.

Another challenge is evaluating AI based on benchmarks and measures that were developed for human agents and for human-human interaction. As a recent study showed, there is a systematic problem with AI benchmarks due to insufficient definitions, problems with internal validity and statistical rigour (Bean et al., 2025). This gains in difficulty when complex ethical and philosophical or psychological concepts are used for measurements. For example, asking whether AI is conscious or empathetic implicitly assumes that consciousness and empathy are well-defined, measurable human properties that can be scaled or replicated. Yet, we lack a unified account of consciousness even in humans (Ferrante et al., 2025). Treating AI as a candidate for human-like consciousness may therefore reflect more about human projection than about AI itself. What is needed is strong construct validity with clear definitions, representative tasks, and sound statistical analysis (Bean et al., 2025). What is also needed is a well-grounded understanding of AI and its role in our practices.

## 3 Ethical human-AI and human-robot interaction and collaboration: relational and sociotechnical account

An alternative account of ethical AI and robotics moves beyond evaluating artificial systems in terms of their similarity to humans and instead focuses on the conditions under which human–AI and human–robot interactions and collaborations become ethically acceptable or problematic. From this perspective, it is more meaningful to talk about ethical AI and robots as always embedded within practices of interaction and collaboration with humans. This perspective makes it explicit that AI and robots are always part of our relationships, necessarily shaped by human goals, meanings and values as well as by broader sociotechnical environments (Coeckelbergh, 2021).

What is at stake, then, is not whether AI or robots possess moral or conscious capacities or other human abilities, but whether the forms of interaction and collaboration they enable lead to harm or support beneficial and meaningful collaborations aligned with the normative expectations of the specific domain in which they are deployed. This shift in focus and also methodology allows ethical evaluation to move away from abstract questions about agents’ properties toward concrete questions about how relations with AI are constituted, governed, and evaluated in practice. Relational accounts of AI ethics emphasize that ethical meaning does not reside in the intrinsic properties of an agent but emerges through situated interactions, shared practices, normative frameworks, and expectations embedded within sociotechnical systems (Coeckelbergh, 2021; Puzio, 2024; Reinecke et al., 2025). From this standpoint, the central ethical questions focus on how particular ways of relating to AI reshape responsibilities, authority, trust, and norms of collaboration. Importantly, these relations are context-dependent: what counts as ethically appropriate interaction differs substantially across domains, such as healthcare, education, or military applications, each of which carries distinct values, vulnerabilities, and risk profiles.

A recent large-scale meta-analysis shows that combining humans and AI does not automatically lead to better outcomes; rather, performance gains depend critically on how interaction is structured, how tasks are allocated, and how coordination between human and artificial agents is designed (Vaccaro et al., 2024). Human–AI systems often fail to outperform either humans or AI alone when interaction is poorly designed. Another study (Zając et al., 2024) situates core ethical principles such as explainability in the sociotechnical environment, showing how requirements for explainability depend on user expertise level, patient context, medical knowledge and clinical type. McCradden and Stedman (McCradden and Stedman, 2024) emphasize that the AI explainability in clinical decision-making must be contextualized. They argue that explainability alone is insufficient for good clinical decisions unless it is situated within broader considerations such as the goals of care, the specific patient and social context, and the clinician’s responsibility to exercise reasonable professional judgment.

These studies underscore that ethical and effective collaboration with AI emerges from understanding relational configuration. Thus, ethical human–robot and human-AI interaction and collaboration cannot be reduced to isolated technical features or to simulation of human characteristics or abilities. It is key to look at the ways AI systems are embedded in social practices, institutions, and meaning-making processes. Ethical design must therefore attend not only to functionality but to how embodiment shapes relational dynamics and how it might expand vulnerability (Tavory, 2024).

Robots, in addition, introduce the dimension of embodiment, which significantly affects ethical interaction (Coeckelbergh, 2021; Nyholm et al., 2023; Torras, 2024). Physical presence can heighten expectations of agency, responsiveness, emotional presence, and care. An embodied robot does not merely produce outputs; it occupies space, moves among humans, and participates in shared environments. These features give rise to distinct ethical considerations that cannot be fully captured by analyses developed for non-embodied AI systems.

## 4 Discussion

The arguments developed in this paper imply that moving beyond the humanization narrative requires interdisciplinary work that pays attention to the relational and sociotechnical dimensions in which AI systems and robots are embedded and the conditions under which their use becomes ethically acceptable. Writing from the perspective of an ethicist, I focus on clarifying how ethics contributes to such collaboration by outlining a structured process that integrates several forms of expertise necessary for imagining, designing and evaluating ethical AI systems. Moreover, I discuss recurring disagreements and tensions between disciplines that arise when integrating ethical analysis.

Interdisciplinary work on ethical AI or robots should begin with a specific use case. Ethical evaluation should not be conducted at the level of AI or robots in general, but must consider particular systems, domains, and forms of interaction. A first step is therefore to identify the purpose of the system, the type of interaction or collaboration it is intended to support, and normative expectations together with key values of the domain. For example, in psychotherapy, ethical interaction relies on norms and values such as trust, honesty, responsibility, or care. Understanding these norms provides the context in which the ethical evaluation of AI systems must take place.

The overall strategy should not focus on AI simulation of human abilities, for example, whether AI systems are empathetic, but on how and to what extent AI systems support or hinder these norms. Ethical evaluation thus shifts from focusing on AI properties to evaluating the role AI systems play within existing relations, practices and networks of values.

When AI is designed with a human-like feature, ethical analysis clarifies the purpose of this feature, how it relates to the domain-specific norms and values, and what weaknesses and strengths AI simulation brings. This analysis requires technical expertise regarding AI design and functioning, which also grounds conceptual and ethical analysis in real applications. This step also examines whether alternative approaches beyond humanization could better support the intended purpose. Ethics provides here conceptual and normative clarification, analyzing which concepts remain relevant and in which sense, how their meaning changes when applied to AI systems, what novelties they bring, where their limits lie and what kinds of conceptual adjustments or alternatives are ethically desirable. Such ethical analysis does not focus on one value or one human-like feature, but places them in a network of values and evaluates the trade-offs that arise between them.

Domain expertise becomes particularly important here. It provides experts' knowledge on normative expectations from the application domain as well as empirical understanding of how interactions function within the domain and whether certain forms of interaction benefit users more than others. This helps assess whether other forms of interaction would better support the intended purpose. For example, forms of companionship may be achieved through interaction models inspired by human–animal relations rather than imitation of human therapists. Insights from fields such as biology, ethology, or the humanities can provide models of coordination, communication, and care that do not rely on imitation of human cognition or emotion. By integrating such perspectives, interdisciplinary work moves beyond the question of whether AI resembles humans toward exploring how it might contribute meaningfully within sociotechnical systems.

Even when technical, domain, and ethical expertise are combined, an important dimension remains: AI systems are designed for end users and the public. Their perspectives must therefore be integrated to ensure that ethical evaluation reflects real-world contexts and expectations. Public engagement is particularly important given that expectations are often shaped by human-centered narratives. Engaging users allows these assumptions to be examined and challenged, while ensuring that those affected by AI systems participate in shaping their development.

At the same time, interdisciplinary collaboration is often challenging. Differences in methods, vocabularies, and epistemic standards can lead to misunderstandings or disagreements between disciplines. Ethics, in particular, is sometimes perceived as too abstract, impractical, or disconnected from real-world applications. Conversely, ethical analysis may question assumptions that are taken for granted in technical design processes or empirical domains.

Several types of disagreement commonly arise in interdisciplinary work. One concerns the role of ethical concepts. Technical research may treat concepts such as fairness, transparency, or trust as measurable properties of systems, while ethical analysis emphasizes that these concepts are embedded in social practices and normative expectations. A second source of disagreement concerns the scope of evaluation. Technical approaches may focus on optimizing specific system features, whereas ethical analysis may highlight broader sociotechnical contexts, including institutional responsibilities, power relations, and long-term societal consequences. A third tension concerns the role of empirical and normative aspects and how they relate to the ethical evaluation. When empirical studies show that there are positive outcomes associated with AI simulating empathy, it does not mean that such simulation is ethically good from normative reasons. This can be illustrated by our societal and ethical attitude towards lying. There could be empirical studies showing that lying pays off, but this does not mean that lying is ethically good.

Addressing these tensions requires practices that enable meaningful collaboration across disciplinary boundaries. While technical and domain expertise help make ethical analysis more grounded and practically relevant, ethics, in turn, contributes by clarifying key concepts, identifying normative expectations, and making explicit the value assumptions that shape AI development and deployment. Approaches such as adversarial collaboration (Kahneman and Klein, 2009; Nature, 2025) and adversarial co-operation (Parker, 2026) provide useful models by treating disagreement as a starting point for joint inquiry.

In this context, disciplines work together to articulate their assumptions, clarify points of disagreement, and jointly examine how system capabilities, domain practices, and normative expectations interact in specific use cases. Tools such as visualizations can support this process by making system behavior, data flows, and value trade-offs visible and open to shared analysis. Rather than forcing consensus, the aim is to make differences in concepts, methods, and normative commitments constructive. In this way, disagreement becomes a resource for refining concepts, identifying hidden assumptions, and exploring alternative design choices.

Importantly, ethical human–AI and human-robot interaction and collaboration should be understood as an evolving process rather than a static checklist. As AI systems enter new domains and reshape social practices and meaning, both technical capabilities and normative expectations change. Sustaining ethical alignment, therefore, requires ongoing interdisciplinary dialogue and reflexivity, rather than one-off ethical assessments. This process demands epistemic humility: recognition of uncertainty, openness to revision, and respect for the limits of any single disciplinary perspective.
